# Biallelic *COL4A2* Variants Associated With Brain Small Vessel Disease and Brain Malformations

**DOI:** 10.1111/cge.70043

**Published:** 2025-08-13

**Authors:** Anees Muhammad, Mohammad Sadegh Shams Nosrati, Alireza Dostmohammadi, Reihaneh Khorasanian, Mariasavina Severino, Morteza Doustmohammadi, Francesca Madia, Siddharth Srivastava, Aisling Quinlan, Dario Paladini, Federico Zara, Marcello Scala

**Affiliations:** ^1^ Institute of Basic Medical Sciences Khyber Medical University Peshawar Pakistan; ^2^ Department of Neurosciences, Rehabilitation, Ophthalmology, Genetics, Maternal and Child Health University of Genoa Genoa Italy; ^3^ Medical Genetics Unit, IRCCS Istituto Giannina Gaslini Genoa Italy; ^4^ Department of Bioinformatics and Computational Biophysics, Faculty of Biology and Centre for Medical Biotechnology (ZMB) University of Duisburg‐Essen Essen Germany; ^5^ Department of Medical Genetics, Faculty of Medicine Shahid Beheshti University of Medical Sciences Tehran Iran; ^6^ Neuroradiology Unit, IRCCS Istituto Giannina Gaslini Genoa Italy; ^7^ School of Medicine Alborz University of Medical Sciences Karaj Iran; ^8^ Department of Neurology Boston Children's Hospital Boston Massachusetts USA; ^9^ Fetal Medicine and Surgery Unit, Department Mother and Child IRCCS Istituto Giannina Gaslini Genova Italy

**Keywords:** brain hemorrhage, brain small vessel disease, *COL4A2*, collagen type IV, cortical malformations, recessive variants

## Abstract

Deleterious variants in *COL4A2*, encoding type IV collagen's alpha‐2 chain, cause heterogeneous cerebrovascular and developmental brain malformations. While many dominant variants are known, biallelic changes are rarely reported. We reported two severe cases: Case #1, an aborted fetus with cerebral calcifications, hemorrhages, periventricular leukomalacia, and cerebellar disruption; and Patient #2, a 2‐year‐old girl with neurodevelopmental impairment, cortical malformations (frontal schizencephaly, polymicrogyria), and reduced white matter volume. Exome sequencing identified a homozygous missense *COL4A2* variant in case #1 and compound heterozygous loss‐of‐function variants (splicing and truncating) in case #2. All variants were rare and predicted to affect protein stability and function *in silico*. Our cases reinforce the association between biallelic *COL4A2* variants and brain small vessel disease, expanding the recessive COL4A2‐related phenotype to include cortical malformations.

## Graphical abstract

Biallelic *COL4A2* variants cause a spectrum of brain abnormalities such as brain small vessel disease (BSVD). We describe two cases—one with cerebrovascular disruption and one with cortical malformations—expanding the recessive phenotype. Protein modeling reveals destabilization by p.(Arg179Cys), reinforcing its pathogenic role and highlighting collagen IV's essential function in brain vascular and cortical development.

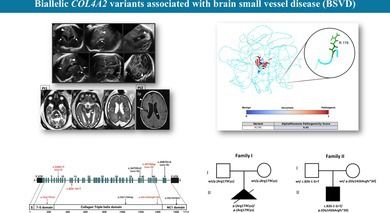

## 1 Introduction

Biallelic variants in *COL4A2* (MIM *120090), encoding the alpha‐2 chain of type IV collagen, are a major determinant of brain small vessel disease (BSVD) and various brain malformations. COL4A2 is a key basement membrane component maintaining structural integrity across tissues, including the CNS [1, 2, 3]. Deleterious *COL4A2* variants are associated with diverse brain anomalies, including cerebrovascular abnormalities and cortical development malformations [2, 4] (Table 1). Recent studies link biallelic *COL4A2* variants to severe neurological disorders (e.g., encephalomalacia, cortical atrophy), suggesting a recessive form and enhancing BSVD knowledge [1, 2, 3]. This complex condition can cause severe manifestations, including recurrent ischemic and hemorrhagic strokes from fetal development into adulthood [1, 2, 3, 5]. Of note, the identification of specific disease‐causing *COL4A2* variants impacting glycine residues shows tissue‐specific gain/loss‐of‐function effects. Glycine is crucial for collagen triple helix formation within the Gly‐X‐Y motif; its substitution by larger amino acids typically disrupts this structure, causing misfolding and functional impairment. This highlights the intricate genotype–phenotype correlation in this disorder [3].

**TABLE 1:** Summary of genetic and clinical features of biallelic *COL4A2* patients.

Cases	Present study		Recent studies			
	Case 1 20‐week‐old fetus	Case 2 2‐year‐old female	PMID: 33912663 8‐year‐old male	PMID: 33912663 20‐year‐old female	PMID: 32040484 18‐year‐old male	PMID: 36603335 7‐years‐old male
Variant (NM_001846.4)	Homozygous c.535C>T, p.(Arg179Cys)	Compound heterozygous [c.826‐1G>T; c.4275dup, p.(Gly1426Argfs*30)]	Homozygous c.3472G>C p.(Gly1158Arg)	Homozygous c.3472G>C p.(Gly1158Arg)	Homozygous c.4987G>A p.(Gly1663Ser)	Compound heterozygous [c.535C>T, p.(Arg179Cys); c 2069G>T, p.(Gly690Val)]
Fetal ultrasonography	Abnormal	N/A	N/A	N/A	N/A	N/A
Brain MRI	Abnormal	Abnormal	Abnormal	Abnormal	N/A	Abnormal
Hemorrhagic necrosis	Yes	Yes	Yes	Yes	N/A	N/A
Porencephaly	No	No	No	Yes	N/A	No
Schizencephaly	No	Yes	No	Yes	N/A	No
Cerebral infarction	No	No	No	No	N/A	No
Leucoencephalopathy	No	No	No	No	N/A	Yes
Hydranencephaly	No	No	No	No	N/A	No
Periventricular leucomalacia	Yes	No	No	No	N/A	No
Motor impairment	N/A	Yes	Spastic quadriplegia, bedridden	Spastic‐dystonic quadriparesis (right‐sided predominance)	Mild gross motor impairment	Yes
Axial hypotonia	N/A	Yes	Yes	Yes	N/A	Yes
Spasticity	N/A	Yes	Yes	Yes	N/A	No
Intellectual disability	N/A	No	Severe, no speech or communicative intent	No speech, globally delayed milestones	No	Yes, delayed language
Epilepsy	N/A	No	Focal epilepsy (onset at 6 months, partially controlled with carbamazepine)	Focal epilepsy (onset at 18 months, controlled with phenobarbital and carbamazepine)	No	Yes, drug resistant
Other findings	Brain calcification, Cerebellar hypoplasia	Polymicrogyria	Cortical visual impairment, ophthalmoplegia, nystagmus, skew deviation	N/A	Bilateral optic nerve hypoplasia	Strabismus, colpocephaly

In this study, we investigated two cases with severe cerebrovascular defects and brain malformations, identifying biallelic *COL4A2* variants predicted to affect protein stability and function. Our findings support the causative role of recessive *COL4A2* in extensive cerebrovascular defects, highlighting that cortical malformations are part of its clinical spectrum [4].

## 2 Case Presentation

This study complies with the Declaration of Helsinki and was approved by the Liguria Regional Ethics Committee (Gaslini Hospital‐163/2018). Written informed consent was obtained from parents or legal guardians for publication of genetic, clinical, and imaging data.

### 2.1 Case #1

**FIGURE 1:**
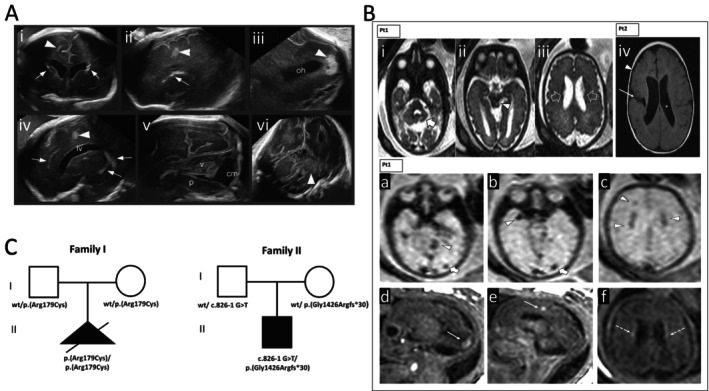
Neuroimaging and genetic features of *COL4A2* patients. (A) Imaging findings, Case #1 Transvaginal neurosonography at 30 gestational weeks: (i–iv) Diffuse perivascular calcifications (arrows) and hyperechoic necrotic white matter lesions (arrowheads) [(i): Coronal transcaudate; (ii–iii) right parasagittal occipital; (iv) left parasagittal]; (v) midsagittal infratentorial view showing vermian (v) and pontine (p) hypoplasia; (vi) coronal transcerebellar view showing severe hypoplasia of the right cerebellar lobe (arrowhead). (B) Fetal brain MRI at 30 weeks in Case #1: (i–iii) Axial T2 images show left‐dominant cerebellar hypoplasia (thick arrow), abnormal foliation, vermian involvement, left midbrain parenchymal loss (arrowhead), and periventricular leukomalacia (empty arrows); (iv) Patient #2: Open‐lip schizencephaly in right posterior frontal lobe (arrow), polymicrogyria extending anteriorly (arrowhead), right ventricular enlargement, and reduced left periventricular white matter (asterisk). (a–c) Patient #1 FMRI, calcifications in the left cerebellar hypoplastic hemisphere, right temporal pole, and periventricular white matter (arrowheads). (d–f) Cortical–subcortical T1 hyperintensities with corresponding T2* hypointensities, partially calcified hemorrhagic infarcts (arrows, thick arrows); small T1 hyperintensities along the irregular ependyma consistent with hemorrhagic periventricular leukomalacia (dashed arrows). (C) Pedigrees, *COL4A2* variants segregation with phenotypes, two families. [Colour figure can be viewed at wileyonlinelibrary.com]

This is a 30‐week‐old fetus conceived by healthy, non‐consanguineous parents. Pregnancy was complicated by intrauterine growth retardation. Fetal ultrasonography (Figure 1A) and brain MRI performed at 30 gestational weeks revealed severe cerebrovascular lesions and cerebellar disruption, leading to a decision of a therapeutic abortion by the parents. Previous pregnancy interrupted at 18 GW for spina bifida.

### 2.2 Case #2

A 2‐year‐old female, born at term (40 weeks, 2.38 kg, −2.21 SDS, SGA) to nonconsanguineous parents after an uneventful pregnancy, required postnatal glucose monitoring. Her neonatal course included perioral cyanosis and spontaneous pneumothorax, necessitating respiratory support. Diagnosed with developmental delay and severe speech impairment in her first year, she babbled at 15 months and vocalized simply by 3 years. At age 2, examination revealed left hemiplegia, dystonia, axial hypotonia, and spasticity in her left limbs with limited arm use. By age 3, she could roll, crawl, and support on forearms/wrists in prone.

## 3 Neuroimaging Studies

In Case #1, fetal brain MRI revealed periventricular leukomalacia (white matter volume loss, enlarged lateral ventricles, irregular ependymal contour). Cortico‐subcortical hemorrhagic lesions were noted in the left occipital and right frontal lobes, alongside multiple periventricular white matter calcifications. A small left midbrain parenchymal loss was visible. Left‐sided cerebellar hypoplasia with abnormal foliation and hemosiderin deposits was consistent with prior hemorrhagic infarct disruption. (Figure 1B,i–iii,a–f).

In Case #2, brain MRI showed open‐lip schizencephaly in the right frontal lobe lined with abnormal gray matter (Figure 1B,iv). The right frontal cortex exhibited irregular thickening consistent with polymicrogyria, characterized by excessive, abnormally structured small gyri. The right lateral ventricle was enlarged and distorted. The periventricular white matter volume was slightly reduced, with consequent mild left lateral ventricle enlargement.

## 4 Exome Sequencing and Variant Analysis

**FIGURE 2:**
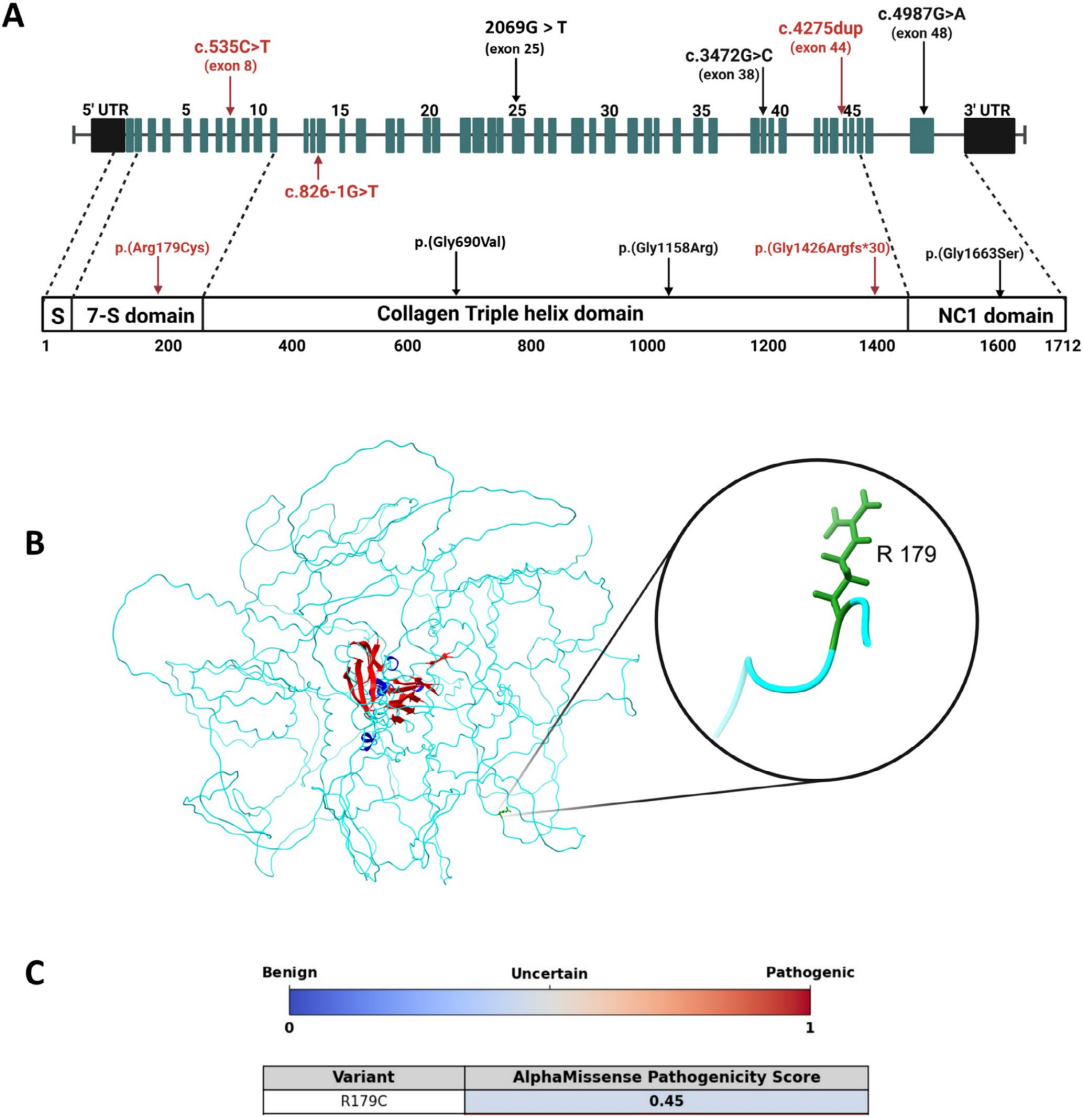
Computational modeling of *COL4A2* variants. (A) Schematic of *COL4A2* gene showing exon‐intron structure, functional domains, and key variants. Exons (dark blue) are numbered; the 7S domain (exons 1–10), the triple helix (exons 11–46), and NC1 (exons 47–48). Pathogenic variants are in red, and previously reported variants are in black. (B) 3D models of mutant COL4A2 showing Arg179Cys substitution. (C) AlphaMissense scores for R179C variant, from benign (blue‐score = 0) to pathogenic (red‐score = 1). [Colour figure can be viewed at wileyonlinelibrary.com]

Exome sequencing (ES) was performed on DNA from affected subjects and their parents; candidate variants were filtered by frequency (gnomAD‐v4.1.0), affected residue conservation (GERP score), and in silico predicted impact on protein structure and function [6]. In Case #1, a homozygous *COL4A2* c.535C>T, p.(Arg179Cys) (exon 8; Figure 2A) was identified, inherited from unaffected heterozygous parents. This very rare variant (AF‐0.00001449‐gnomAD), absent in homozygous healthy individuals, affects a highly conserved residue (GERP = 5.33) and is predicted to be moderately damaging, classified as likely benign (ACMG Class‐II; BP1, BP4). For Case #2, two compound heterozygous variants were detected: paternal c.826‐1G>T (intron 13) and maternal c.4275dup (exon 44), p.(Gly1426Argfs*30) (Figures 1C and 2A). Both are rare and predicted to be loss‐of‐function. The splice‐site c.826‐1G>T disrupts the acceptor site at −1, classified as likely pathogenic (ACMG Class‐IV; PVS1). The p.(Gly1426Argfs*30) is predicted to cause loss of function due to the formation of a truncated transcript or nonsense mediated mRNA decay (NMD), also classified as likely pathogenic (ACMG‐PVS1, PP5). All variants are reported using NM_001846.4 transcript.

## 5 Protein Modeling of the Missense p.(Arg179Cys) Variant

The three‐dimensional structure of COL4A2 has not been experimentally elucidated. We employed AlphaFold2 to forecast its structure [7]. The anticipated model may be accessed in the AlphaFold Protein Structure Database with the identifier AF‐P08572‐F1. To assess the impact of the p.(Arg179Cys) variant on protein stability, we utilized ThermoMPNN [8], a deep neural network developed on extensive stability data, leading to a result of 0.1296 kcal/mol. Then, we used Rosetta (version‐3.14) [9], which employs a synthesis of physics‐based and statistical energy functions, leading to a result of 10,719.264 Rosetta Energy Units (REU) compared to the wildtype protein (9251.946 REU). Overall, stability evaluations demonstrated that the R179C destabilizes the COL4A2 protein structure (Figure 2B). Furthermore, we employed AlphaMissense to predict the pathogenicity of the missense change, which resulted in a score of 0.45 [10] (Figure 2C).

## 6 Discussion

The *COL4A1* and *COL4A2* genes encode the alpha‐1 and alpha‐2 chains of type IV collagen, essential components of basement membranes. The alpha‐2 chain is unique due to a 21‐amino acid disulfide‐bridged loop within its triple helix [11, 12, 13]. Dominant‐negative or haploinsufficient variants in these genes are recognized to lead to a multisystem microangiopathy primarily affecting the brain (recurrent ischemic/hemorrhagic strokes, porencephaly, leukoencephalopathy), alongside ocular, renal, cardiac, and muscular systems [2, 13, 14, 15, 16, 17, 18].

Both *COL4A1*/*COL4A2* variants are associated with ICH, congenital porencephaly, and severe fetal cerebral lesions [13, 14, 17, 18]. COL4A1‐related disorders typically present with more severe and penetrant features, compared to *COL4A2*‐associated conditions, often with a dominant inheritance and variable expressivity, including frequent congenital porencephaly, perinatal intracerebral hemorrhage, ocular anomalies, and systemic involvement [2, 14, 15, 16, 19, 20, 21, 22]. Even without overt porencephaly/stroke, subclinical findings including periventricular leukoencephalopathy, intracranial calcifications, aneurysms, and microbleeds underlie a broad cerebrovascular disease (CVD) spectrum [14]. Extra‐CNS involvement is also common in COL4A1 patients, featuring ocular anomalies (e.g., cataracts, anterior segment dysgenesis, retinal vascular tortuosity), intracranial calcifications, and myopathy mimicking congenital muscular dystrophy or Walker‐Warburg syndrome [20, 23, 24, 25].

In contrast, *COL4A2* variants often show slightly less severe cerebrovascular phenotypes, such as BSVD or adult‐onset hemorrhagic stroke, with lower penetrance [13, 15, 17]. This difference likely stems from chains' distinct but interdependent roles in basement membrane integrity and vascular stability [14, 15, 16]. A different scenario occurs in the case of biallelic *COL4A2* variants, which are exceptionally rare. These substitutions have been first identified in 2012 in association with severe disorders, such as ICH and porencephaly [13, 16], and later linked to encephalomalacia and cortical atrophy [1, 2, 3, 4]. Our findings support the association of recessive *COL4A2* variants with more severe clinical presentations compared to monoallelic substitutions. Case #1 showed diffuse calcifications, cerebellar hemorrhage, and cortical/subcortical necrosis, consistent with BSVD. Case #2 presented severe malformations of cortical development, likely reflecting disrupted cortical development and vascular instability [1, 2, 3, 26]. As such, these extreme phenotypes underscore a highly deleterious clinical impact of recessive variants, comparable to or even exceeding *COL4A1*‐related presentations. If confirmed by further reports, this may challenge our current view on *COL4A2*‐related disorders, suggesting that they can manifest as a broad range of cerebrovascular phenotypes with highly variable severity [15].

Our study expands the *COL4A2* genotype spectrum by identifying two novel variants. In case #2, we detected the frameshift variant p.(Gly1426Argfs*30) within the triple helix domain, and a splice‐site variant, c.826‐1G>T. The frameshift is predicted to severely destabilize protein structure, leading to misfolding and loss of function, consistent with other collagenopathies [27]. The splice‐site variant likely disrupts mRNA splicing. Both variants are predicted to significantly impair COL4A2 expression and function. In case #1, we identified the homozygous p.(Arg179Cys) variant, which has been recently reported *in trans* with the p.(Gly690Val) variant in a patient with leukoencephalopathy [2]. This non‐glycine variant affects a conserved residue within the N‐terminal region external to the triple helix domain, a region less commonly associated with human disease, likely implicating abnormal protein folding or assembly.

Most pathogenic *COL4A1*/*COL4A2* variants affect Glycine (Gly) residues within the triple helical domain. Glycine's small side chain is critical for proper collagen triple helix folding [14, 15, 19, 27]. Substitution by larger amino acids disrupts helical conformation, leading to impaired trimer formation, intracellular retention, or basement membrane instability [14, 27]. These dominant‐negative substitutions typically cluster in conserved triple helical regions, correlating with disease severity and multi‐systemic involvement [13, 14, 15, 16, 17, 18]. Non‐glycine *COL4A2* variants (e.g., E1123G, Q1150K in Gly‐XY; A1690T in NC1) are known to cause decreased COL4A2/COL4A1 secretion via ER retention, leading to ICH [16]. Such examples, including p.(Gly1663Ser), impact the NC1 domain's stoichiometry [3]. This highlights diverse pathogenic mechanisms beyond canonical Gly‐X‐Y disruptions. In our case, Alpha Missense predicts that the p.(Arg179Cys) highly destabilizes protein structure, suggesting that it may act as a hypomorphic allele in causing disease.

In conclusion, our study emphasizes the critical role of biallelic *COL4A2* variants in BSVD and severe brain malformations, significantly expanding the genotype and phenotype spectrum of recessive COL4A2‐related disorders. These findings support that recessive *COL4A2* variants can cause a broad spectrum of highly penetrant and severe cerebrovascular abnormalities.

## Author Contributions

A.M. and M.S.S.N. designed the study, literature review, and drafted the manuscript. A.D. and R.K. contributed data analysis. M.S. and M.D. conducted neuroimaging review. F.M. supported variant analysis. S.S., A.Q., D.P., and F.Z. revised the manuscript. M.S. supervised the study and is the corresponding author. All authors approved the final version.

## Conflicts of Interest

The authors declare no conflicts of interest.

## Data Availability

The data that support the findings of this study are available from the corresponding author upon reasonable request.
